# Cost-effectiveness of population-based expanded reproductive carrier screening for genetic diseases in Australia: a microsimulation analysis

**DOI:** 10.1038/s41431-025-01835-8

**Published:** 2025-04-16

**Authors:** Deborah Schofield, Evelyn Lee, Jayamala Parmar, Adriana Castelo Taboada, Matthew Hobbs, Nigel Laing, Rupendra Shrestha

**Affiliations:** 1 Centre for Economic Impacts of Genomic Medicine, Macquarie University, Macquarie Park, NSW Australia; 2 Leeder Centre for Health Policy, Economics & Data (The Leeder Centre), University of Sydney, Sydney, NSW Australia; 3 Computational Biology, Data Science Platform, Garvan Institute of Medical Research, Darlinghurst, NSW Australia; 4 Centre for Medical Research University of Western Australia, Nedlands, WA Australia

**Keywords:** Health care economics, Health policy

## Abstract

Using the Australian Census survey 2021 as base population, a microsimulation model, *PreconMOD* was developed to evaluate the cost-effectiveness of population-based expanded reproductive carrier screening (RCS) for 569 recessive conditions from the health service and societal perspectives. The model simulated the effect of expanded RCS including the downstream interventions for at-risk couples on cost and outcomes. The comparators were (i) no population screening (ii) limited screening for cystic fibrosis, spinal muscular atrophy, and fragile X syndrome and (iii) a 300 conditions screening panel. Averted affected births and health service cost with expanded RCS were projected to year 2061. At a 50% uptake, our model predicts that expanded RCS is cost saving (i.e., higher quality-adjusted life-years and lower costs) compared with other screening strategies in the model from the health service and societal perspectives. The number of affected births averted in a single cohort would increase from 84 [95% confidence interval (CI) 60–116] with limited screening to 2067 (95%CI 1808–2376) with expanded RCS. Expanded RCS was cost-saving compared to the 300-conditions screening panel. Indirect cost accounted for about one-third of the total costs associated with recessive disorders. Our model predicts that the direct treatment cost associated with current limited 3 genes screening would increase by 20% each year to A$73.4 billion to the health system by 2061. Our findings contribute insights on the cost burden of genetic diseases and the economic benefits of expanded RCS to better informed resource allocation decisions.

## Introduction

Mendelian disorders are individually rare but the collective burden and cost of treatment for these diseases are considerable [1]. Previous research suggests that the lifetime cost attributable to 300 genetic diseases was A$2.1 billion to society [2]. Carrier screening aims to identify couples at risk of transmitting an inherited disorder to enable consideration of reproductive decisions to avoid passing on the condition.

In Australia since the 1970 s, carrier screening has primarily been targeted to individuals with a known family history of a specific genetic disorder or those from specific at-risk ethnic groups e.g., screening Ashkenazi Jews for Tay-Sachs and people of African or Asian descent for hemoglobinopathies [3]. This targeted screening has been effective in reducing the burden of recessive diseases in Australia and globally. For example, a clinical audit of Tay-Sach Disease (TSD) cases diagnosed between 1995 and 2011 found no incidence of TSD in the Australian Jewish community following the targeted preconception genetic screening programs while screening for fragile X saw a 26% reduction [3, 4].

However, with the complexities of race, ethnicity and coupling pattern, using race or ethnic classifications to estimate genetic risk for panel selection has been increasingly challenging resulting in an increased likelihood of disease occurrence in non-targeted groups [5]. This has been shown in several studies including a recent retrospective review of clinical data of 23,453 individuals for 108 genetic disorders where almost one in four pregnancies had at least one disorder that is not covered by current screening guidelines [6, 7].

With growing evidence supporting the benefits of a pan-ethnic approach to carrier screening and the falling cost of sequencing, professional organizations such as the American College of Obstetricians and Gynaecologist (ACOG) endorse expanded carrier screening for genetic diseases for individuals who are considering pregnancy or currently pregnant irrespective of their reported ethnic background [8]. Similarly, the American College of Medical Genetics and Genomics (ACMG) updated its recommendations to support pan-ethnic carrier screening for 112 conditions [9].

However, to the payer, the cost of screening is an important consideration. Previous modelling studies have shown that expanded RCS (reproductive carrier screening) for 300 conditions offered at the population level is cost-effective from healthcare system and societal perspectives [2, 10, 11]. One study showed that at an uptake rate of 50%, expanded RCS for a 300 conditions panel would avert 2290 more affected births in a single birth cohort and reduce lifetime health service cost associated with the affected births by A$632.0 million (in 2021) compared to limited screening for only three common conditions - spinal muscular atrophy (SMA; OMIM #253300), cystic fibrosis (CF; OMIM#219700) and fragile X syndrome (FXS; OMIM#300624) [2].

With improved efficiency and declining genomics costs, there has been a rapid proliferation of commercial companies offering RCS, with panel size ranging from three to 2054 genes and varying number of genes with different carrier frequency, mild or incompletely degree of penetrant phenotypes [12]. As more genetic variants and rare conditions are increasingly offered on screening panels, it is important to know whether the benefits of offering expanded screening with more conditions is cost-effective from the payer perspective. Indeed, in most countries, such as Australia, carrier screening beyond the most common conditions is not publicly funded and requires a substantial patient copayment that may discourage screening uptake.

Therefore, this study assessed whether expanded RCS provides value for money from the health system and societal perspectives. Specifically, our study evaluated the cost-effectiveness of a commercially available RCS consisting of a 569 autosomal recessive gene panel (hereafter referred to as ‘569 conditions expanded RCS’) relative to (i) a 300 autosomal recessive gene panel (hereafter referred to as ‘300 conditions screening panel’). The study also examined the cost-effectiveness of expanded RCS for 569 conditions with (ii) no population screening and (iii) preconception and prenatal population screening of three common conditions ─ CF, FXS and SMA (hereafter referred to as limited screening’). In this analysis, we included outcomes of the downstream interventions following expanded RCS on the overall life-time cost and outcomes (life-years or cases averted) in a single birth cohort and projected costs and outcomes to 2061 (over 40 years) to better inform resource allocation decisions.

## Materials and methods

### Modelling approach

Details of *PreConMOD* to evaluate the cost and outcomes of expanded RCS was described elsewhere [2]. Briefly, from the inception all prospective parents were offered a 569 conditions screening panel, and the model simulated their progress through the pathway from their first decision to undertake (or decline) expanded RCS, and the downstream interventions for at-risk couples who wanted to avoid having affected children.

Age specific mortality data was derived from the Australian Bureau of Statistics (ABS) life tables for year 2022. Rates were converted to probabilities. Transitions through the model were determined using a stochastic process with random samples drawn based on model parameter distributions and assigned probabilities leading to different simulation outcomes for each at-risk couple (Supplementary Table 1).

### Base population

The base population of *PreConMOD* consists of 1% census data of which families with children <1 year of age were used to estimate the number of children born in the previous year. In our study, there were 309,996 families with newborns in the 2021 Australian population after adjusting for undercounting due to reasons including delay in registration of births, failure to complete the Census form and deaths of children born during the year prior to the census.

### Reproductive choices for at-risk couples

We modelled access to publicly funded assisted reproductive treatment (ART) strategies where at-risk couples undertake up to two cycles of ART with pre-implantation genetic testing (PGT) or donated gametes during in vitro fertilisation (IVF) treatment which could either result in pregnancy leading to a live birth or no pregnancy. These strategies were configured to represent the likely clinical pathway of Australian couples seeking ART in real-world settings [13]. However, at-risk couples may also continue with natural conception or choose not to have children.

For at-risk couples undertaking PGT, the model started with a pathogenic variant carrier undertaking one cycle of IVF and PGT which could either result in pregnancy leading to a live birth or no pregnancy. Where no pregnancy was achieved with ART, at-risk couples may also continue with natural conception or choose not to have children.

Similarly, for an at-risk couple using ART donated gametes, the model started with a pathogenic variant carrier undertaking one cycle of IVF using donated gametes where it could either result in pregnancy leading to a live birth or no pregnancy. Where no pregnancy was achieved, we modelled undertaking IVF and PGT, not having children or continuing with natural conception.

For those who achieved pregnancy after natural conception or had already conceived when they received screening results, we modelled access to prenatal diagnostic testing and the probability of termination of pregnancy if the foetus is affected. Estimates of transition probabilities between the different reproductive choices were retrieved from published literature [13].

### Disease list

Supplementary Table 2 contains the full gene list which was based on a widely available commercial expanded carrier screening panel covering 569 autosomal recessive and X-linked conditions aimed at identifying carriers of a genetic condition that could affect their offspring in Australia (Invitae Comprehensive Carrier Screen, Invitae, San Francisco, CA). However, at the time of writing, Invitae has filed for bankruptcy and sold their reproductive health assets to Natera [14].

### Data sources

For each of the 569 recessive disorders in the expanded panel, model inputs were derived using published studies, reports, and judgments from an expert panel. We also utilised web-based resources such as OMIM, Orphanet and National Organisation for Rare Disorders to develop a list of keywords that best define the phenotype associated with gene-related disorders before searching via OVID MEDLINE for quantitative evidence. These model inputs were incidence rate, age of disease onset, age of death, Quality Adjusted Life Year (QALY), life expectancy, direct and indirect costs for each of the 569 recessive disorders included in the expanded RCS panel. We also reviewed reference lists of relevant articles to ensure significant publications on key evidence were not missed. Where information was lacking, they were obtained from other related diseases with similar attributes for which data was available.

### Model validity

In line with best practice recommendations for model validation [15], experts in clinical genetics, modelling and health economics critically reviewed the model structure and logic, and reviewed model outcomes to ensure face validity.

### Utility (QALY) and costs outcomes

For all models, age- and sex-specific utility scores for the “disease free” health state were extracted from a cross-sectional Australia-specific study (*n* = 2900 healthy individuals) measuring quality-of-life scores for the general population [16].

### Costs

We performed the economic analysis in two parts. The first part of the economic analyses involved modelling the costs of up-front carrier screening, subsequent downstream interventions following RCS including fertility treatments, prenatal diagnostic test and termination of pregnancy. The downstream costs were obtained from the Australian Medicare Benefits Schedule where applicable (Supplementary Table 3).

The cost of carrier screening was modelled based on the commercial list price in Australian dollars A$805 per couple screening for a 569-condition expanded RCS panel. This price is comparable to the average commercial list for expanded carrier screening available in the Australian market [12].

The second part modelled the lifetime disease treatment costs and indirect costs associated with each of the 569 recessive disorders included in the expanded RCS panel in the absence of carrier screening. For most resources such as disease treatment costs, they were obtained from the literature. In this study, costs reflect the resources required to deliver the respective interventions as well as the resources required for treatment associated with genetic disease. All costs were adjusted to 2021–2022 Australian dollars using the AIHW health inflation index [17].

### Quality adjusted life year (QALY)

The main outcome of interest was QALYs. QALYs are a composite measure of health which combine the length of time spent in a health state by the utility associated with the health state. Utilities are measured on a scale of 0 to 1, where 0 represents a health state equivalent to death and 1 represents a health state of perfect health. Therefore, one year spent in perfect health is equal to 1 QALY and 1 year spent at a utility of 0.5 is equivalent to 6 months with perfect health. The use of QALYs is intended to provide a single metric to measure health outcomes and thus allow for comparisons of alternative interventions regardless of disease/condition and the consideration of opportunity cost across the entire health care system [18]. In this study, health utility estimates associated with each gene on the RCS panel were obtained from the literature.

Other outcomes associated with each of the strategies were estimated based on the number of affected births averted and years of life as a result of carrier screening and reproductive decisions (e.g., IVF and PGT).

### Comparators

We assessed the outcomes of a single birth cohort of couples who undertook expanded RCS for 569 conditions panel with three comparators:

(i) a counterfactual birth cohort of parents that has never had carrier screening (i.e., no population screening);
(ii) a second counterfactual single birth cohort of parents who had been offered limited screened for only 3 conditions– CF, SMA and FXS (i.e., the “3-genes screening panel”)
(iii) a third counterfactual single birth cohort of parents who had undertaken screening for 300 recessive conditions only (i.e. the “300-conditions screening panel”).

### Cost effectiveness analysis

A cost-effectiveness analysis was conducted using overall life-time costs and outcomes (i.e., QALY, life years). Incremental cost-effectiveness ratios (ICERs) were calculated by dividing the difference in costs by the difference in QALYs between the expanded RCS and the three comparators- (i) no carrier screening; (ii) limited 3-genes screening panel and (iii) a 300-conditions screening panel.

We simulated the outcomes that occurred over the lifetime of a single birth cohort by comparing the three alternative screening strategies with expanded 569 conditions panel. Incremental cost-effectiveness ratios (ICERs) were calculated using the incremental life-time costs and outcomes (i.e., QALY) between the expanded 569 conditions panel and the three comparators. Total costs included cost of one-off carrier screening, subsequent downstream interventions, and lifetime disease treatment costs associated with each of the 569 recessive disorders in the expanded panel and the three comparators.

### Sensitivity analyses

To address the inherent uncertainties surrounding our assumptions on model parameters, one-way sensitivity analysis was performed for key parameters including screening uptake rate, market price for RCS and termination of affected foetus to characterise the impact of varying these parameters on the ranking of strategies in the cost-effectiveness analysis.

Probability sensitivity analyses were conducted using Monte Carlo simulation with 1000 iterations, sampled from the probability distribution around the variables’ mean values for each of the 569 genes in the panel with accompanying 95% confidence intervals (95% CIs). We assumed beta distributions for all probabilities and utilities whose values were bounded between 0 and 1 and gamma distributions for costs to capture its non-negative and skewed features.

All analyses were undertaken from both health service and societal perspectives in the Australian setting. Expanded RCS for 569 conditions was considered cost effective if the additional cost per QALY gained was less than the threshold of a willingness-to-pay (WTP) of A$50,000, a widely used indicative threshold for public funding in Australia. The total discounted lifetime costs and life-years were calculated by accruing the predicted costs and life-years from birth to death and discounting at an annual rate of 5% as per Australian guidelines [17].

## Results

### Base case results

- No population screening.
  Our model predicts that in the absence of population-based reproductive carrier screening, about 7623 Australian children would be born with autosomal recessive disorders based on the total incidence rates for 569 inheritable recessive disorders in the screening panel in a birth cohort. The total estimated lifetime treatment costs attributed to the recessive conditions in a single birth cohort of children in 2021 would be A$1.87 billion to the healthcare system (Supplementary Table 4).
- 569-condition screening panel versus limited 3 gene screening panel.
  Our model estimated that offering population-based screening for 569 conditions and subsequent downstream interventions would cost A$120.8 million more than the limited 3 genes panel. Incremental cost-effectiveness analysis (ICER) results for the expanded 569 condition panel under base case assumptions are shown in Table 1.

**Table 1:** Cost effectiveness of population-wide expanded preconception carrier screening.

	Up-front carrier screening A$M	Downstream intervention costs, A$M	Total Investment, A$M	Life-years, years	Total cases averted, n	Total cost A$B	Total QALY ‘000	ICER with expanded PCS (A$)
No population screen	—	—	—	113,253 (97,021 to 130,700)	—	1.87 (1.58 to 2.20)	68.9 (60.9–77.4)	
Expanded 569 RCS	146.6	125.3	271.9	102,817 (88,773 to 118,016)	2067 (1808 to 2376)	1.70 (1.51 to 1.92)	78.9 (68.6–91.2)	Cost savings (lower costs and higher QALY)
Three -genes screening panel	145.7	4.4	150.2	110,874 (94,764 to 127,931)	84 (60 to 116)	1.71 (1.5 to 1.94)	69.5 (61.0–78.7)	
Expanded 569 RCS	146.6	125.3	271.9	102,817 (88,773 to 118,016)	2067 (1808 to 2376)	1.70 (1.51 to 1.92)	78.9 (68.6–91.2)	Cost savings (lower costs and higher QALY)
300 -conditions screening panel	146.6	71.9	218.5	103,323 (89,284 to 118,189)	1432 (1203 to 1722)	1.76 (1.5 to 19.1)	78.3 (67.8–89.8)	
Expanded 569 RCS	146.6	125.3	271.9	102,817 (88,773 to 118,016)	2067 (1808 to 2376)	1.70 (1.51 to 1.92)	78.9 (68.6–91.2)	Cost savings (lower costs and higher QALY)

RCS: Reproductive carrier screening; Cost in A$ 20211-2022 values. The additional healthcare savings was due to cases prevented compared to respective screening strategy. Three genes  screening panel refers to combined screening panel for cystic fibrosis, spinal muscular atrophy and Fragile X syndrome. Downstream intevention included reproductive options (IVF in combination with PGT or donated gametes), prenatal diagnostic testing and termination of pregnancy.

Incremental cost-effectiveness ratio (ICER) refers to the difference in the costs between expanded RCS and three-genes screening panel or no population screening. Expanded RCS is cost-saving when it is associated with lower costs and improved QALY compared to respective strategy in the model. 95% confidence interval in the parenthesis were calculated using 1000 bootstrapping samples (probability sensitivity analyses). Abbreviation: *Expanded RCS* Expanded reproductive carrier screening, *QALY* Quality-adjusted Life-years, *A$M* Million in Australian dollars; *A$B* Billion in Australian dollars.
  Our model predicted that screening for 569 conditions would result in a lifetime QALY gain of 78,974 (95% 68,649—91,242), compared to a lower QALY gain of 69,536 (95%CI 61,049—78,799) with limited 3-genes screening panel (based on a 50% uptake rate). Similarly, our model estimated that 2067 (95% 1808—2376) affected births would be averted in a single birth cohort with population-based screening for 569 conditions panel compared to 84 affected births averted (95% 60—116) with limited 3-genes screening panel.
- Expanded 569 RCS -cs versus 300-conditions screening panel.
  A higher lifetime QALY gain and a lower cost was estimated for the expanded condition panel compared to the 300- conditions screening panel. Our model predicts that population based screening for 569 conditions would avert 636 more affected births than the 300 conditions screening panel, resulting in a lower lifetime health service cost of A$1.70 billion (95% A$1.51—A$1.92 billion) for the 569-conditions panel compared to A$1.76 billion (95% A$1.57 billion—A$1.97 billion) for the 300 conditions screening panel (based on a 50% uptake rate). 
- Projected births with recessive disorders for the period 2021 to 2061.

**Fig. 1:**
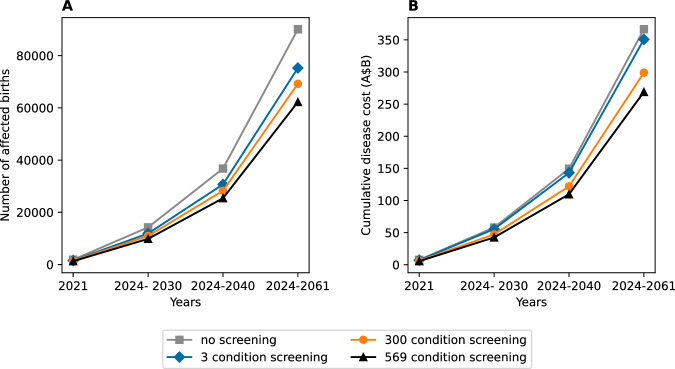
Base case estimate of children born with recessive disorders (using  a 569 recessive genes panel), 2021 and 2024 to  2061, for the four screening strategies. **A** Projected cumulative number of affected births; (**B**) total lifetime healthcare costs associated with recessive disorders. Three-genes screening refers to combined screening panel  for cystic fibrosis, spinal muscular atrophy, and fragile X syndrome in a single birth cohort. Three hundred conditions refers to a 300 recessive genes panel and 569 conditions refers to a 569 recessive genes panel.

Figure 1 shows the overall projected births with recessive disorders based on   different screening strategies in the model over a 40-year period.

Using the ABS projected births for 2021–2061, our base case analyses predict that, 7294 more Australian children will be born with genetic diseases annually under the current limited 3 gene panel. The number of children born with recessive disorders was estimated to increase to 143,083 in 2040 and 350,715 by 2061. The mean lifetime cost burden for children born with genetic disease was estimated to grow by 20% each year to A$74.8 billion in higher direct costs by 2061 if carrier screening was limited to the 3 gene panel. However, a 569-condition expanded RCS is estimated to avert 2170 more affected births compared to other screening strategies in the model.

**Fig. 2:**
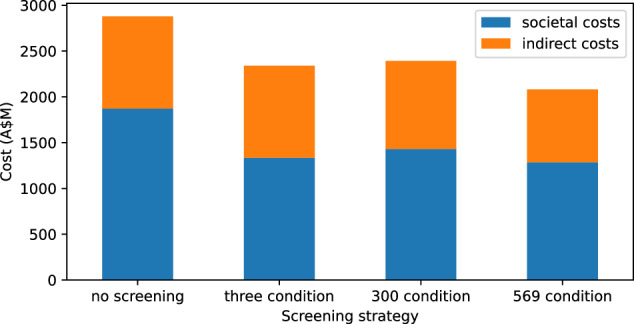
Indirect and societal costs attributed to a 569 conditions panel. Societal cost refers to out of pocket costs associated with reproductive services (e.g., IVF) and the cost of reduced labour force productivity due to caring for affected individuals with a genetic disease. Three- genes screening refers to combined screening  for cystic fibrosis, spinal muscular atrophy, and fragile X syndrome in a single birth cohort. Three hundred conditions refers to a 300 recessive genes panel and 569 conditions refers to a 569 recessive genes panel.

Our models showed that the indirect cost borne by individuals accounted for slightly more than one-third of the total costs associated with recessive disorders (35%) (Fig. 2). When taking into account both healthcare and indirect costs in a single birth cohort, our model predicts that compared to the limited 3 genes screening panel, the 569-condition expanded RCS was predicted to be cost saving with a mean cost saving of A$227.7 million. Similar cost-saving outcomes were observed when compared with a 300-conditions screening panel.

### Sensitivity analyses

**Fig. 3:**
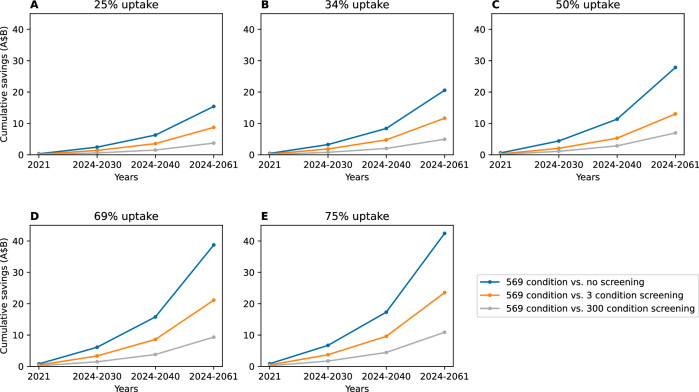
Sensitivity analysis varying uptake rate for expanded reproductive carrier screening. Cumulative cost savings in A$ 2021, 2024-2061 comparing 569 condition screening with three other screening strategies. The cost savings were due to affected births averted. Cost savings based on uptake rates of expanded RCS set at 25% (**A**), 34% (**B**), 50% (**C**), 69% (**D**) and 75% (**E**). A$B Billion in Australian dollars.

Figure 3 presented the results from the one-way sensitivity analysis. Compared to no population-based screening, at 25% uptake, the projected total healthcare savings due to affected birth averted to 2061 was A$15.3 billion and increased to A$42.3 billion when uptake increased to 75%. Increasing the price for expanded screening per couple by 75% would render expanded RCS not cost-effective at willingness-to-pay threshold of A$50,000 per QALY in Australia (Table 2).

**Table 2:** Sensitivity analysis - varying price of expanded preconception carrier screening.

	Up-front carrier screening A$M	Downstream intervention costs, A$M	Total investment A$M	Total costs A$B	Total QALY ‘000	ICER with expanded PCS (A$)
No population screen	—	—	—	1.87	68.9	
Expanded RCS (−75%)	109.8	125.3	235.1	1.66	78.9	Lower cost and higher QALY
Expanded RCS ( + 75%)	256.7	125.3	382.0	1.81	78.9	Lower cost and higher QALY
Three gene condition screening	145.7	4.4	150.1	1.71	69.5	
Expanded RCS (−75%)	109.8	125.3	235.1	1.66	78.9	Lower cost and higher QALY
Expanded RCS ( + 75%)	256.7	125.3	382.0	1.81	78.9	9718 per QALY
300 conditions screening	146.6	71.9	218.5	1.76	78.3	
Expanded RCS (−75%)	109.8	125.3	235.1	1.66	78.9	Lower cost and higher QALY
Expanded RCS ( + 75%)	256.7	125.3	382.0	1.81	78.9	68,377 per QALY

Three gene condition screen refers to combined screening panel for cystic fibrosis, spinal muscular atrophy and Fragile X syndrome. The price of expanded RCS per couple was varied between ± 75% of the base list price at A$805 (US$554 per couple as at November 2023).

Incremental cost-effectiveness ratio (ICER) refers to the difference in the costs between expanded RCS and three-gene condition screening or no population screening. Expanded RCS is dominant when it is associated with lower costs and improved QALY compared to respective strategy in the model.

*RCS* Reproductive carrier screening, *A$M* Million in Australian dollars, *A$B* Billion in Australian Dollars, *QALY* quality-adjusted life years.

## Discussion

Using a microsimulation model, this study evaluated the cost-effectiveness of population-based expanded RCS for 569 conditions and projected the potential costs and outcomes to 2061 in Australia. Our model predicts that in the absence of a population level carrier screening for genetic disease for 539 conditions, about 7623 Australian children would be born with autosomal recessive disorders each year. The total lifetime treatment costs attributed to these disorders for a single birth cohort is estimated to be A$1.87 billion to the health system.

At an uptake rate of 50%, our model predicts that expanded RCS for 569 conditions is cost-saving compared with the three screening strategies - no population screening, limited 3 gene screening panel and the 300-conditions screening panel. Notably, expanded RCS results in higher QALYs gained compared with other screening strategies. The number of affected births averted in a single cohort increased to 2067 with expanded RCS for 569 conditions when compared to 84 with the limited 3-gene screening panel.

Our finding extends the existing knowledge on the cost effectiveness of population-based expanded RCS. Most cost studies have been limited by focusing on direct costs associated with one or a small number of autosomal recessive disorders and short-term impact of carrier screening [7, 10, 11]. Other studies discussed the clinical utility of expanded RCS without explicitly addressing cost [19, 20]. Cost is an important consideration. Despite the current recommended guidelines, expansion of commercial screening panels has largely been driven by advancing technology and declining sequencing cost [12]. This raises questions about the cost-effectiveness of expanded panel with a broader array of genetic variants.

Moreover, owing to the complexity of categorising conditions for inclusion in an ECS panel, different providers may have different views on whether a particular disorder should be characterized as severe, and how genotype–phenotype correlation is defined [21]. There have been concerns that the inclusion of genes with unclear clinical significance could pose a psychosocial burden to prospective parents [20]. Furthermore the cost to couples for accessing expanded RCS increases with the number of genes screened [12]. These barriers have also shown to be more pronounced among couples residing in regional and rural areas where access to RCS screening programs and care is limited [22]. In countries without public funding, financial barriers to access of expanded screening could exacerbate inequalities in the long term. This is important as couples with the fewest resources to manage the cost of caring for an affected child are also the least likely to be able to access RCS testing which is inconsistent with the equity and access aims of the Australian Medicare [22].

Indeed, studies have found that RCS is more accessible to those living in the most advantaged areas highlighting a concerning socio-economic gradient in screening availability and uptake for preconception services [23]. While the Australian government provides public funding for the provision of some reproductive options (IVF with PGT) and prenatal diagnostic testing for at-risk carrier couples, expanded RCS for genetic disease remained limited to those who can afford it [24, 25]. However, in a recent nationwide study by Kirk and colleagues – the Australian Reproductive Genetic Carrier Screening Project (Mackenzie’s Mission), the study reported a nearly 50% uptake rate for expanded RCS among ethnically, socioeconomically diverse and geographically dispersed population when offered. It is estimated that the uptake rate would likely increase if expanded RCS is universally offered and publicly funded [26].

Using microsimulation techniques, our study incorporates all downstream consequences to model the cost and outcomes of 569 conditions expanded RCS to inform and support funding decisions. Our approach overcomes the limitations in previous economic model that did not model uptake rate for RCS and used a single cost estimate for all downstream interventions (e.g., PGT, IVF) which may underestimate cost-effectiveness findings [7, 11]. Furthermore, our study reported outcomes using QALY as indicator—an outcome measure that combines the duration and quality of life is regarded as the gold standard methodology in economic evaluation. The estimates of QALY allows comparison of health benefits across different healthcare interventions in other areas to support priority setting and resource allocation decision [18].

However, as with any modelling, our study relies on key assumptions due to the varying quality of evidence and considerable uncertainty associated with rare conditions. As some diseases evaluated in our model are relatively rare, current knowledge about the long-term costs associated with morbidity are limited. However, we addressed the limitation by setting a wide range of values on the parameters in probability sensitivity analyses. We have modelled the rate of elective termination conservatively at 50% as done in previous studies [2, 10]. This is lower than the Australian Government Medical Services Advisory Committee (MSAC) application #1637 which reported a rate of 67% based on a small sample of 4 out of 6 affected pregnancies [27]. The model assumed perfect sensitivity and specificity for screened diseases as has been done in other studies [11].

Notwithstanding its limitations, our model demonstrates that expanded reproductive carrier screening for recessive disorders has the potential to generate considerable healthcare saving, increase QALY gains and reduce costs to society. Further research should consider the impact of recent genetic technologies including next-generation sequencing and the potential cascading effects of RCS on family members (close relatives) as well as access to genetic counselling and psychological support for patients accessing RCS so that these outcomes can be accounted for in decision making.

In conclusion, our model showed that at a 50% uptake rate, a 569-conditions expanded screening panel is cost-effective and averted significantly more affected births compared to other screening strategies in the model from the healthcare and societal perspectives.

## Supplementary information

Supplementary Tables

## Data Availability

The authors confirm that all data for parameters is fully available without restriction and the Census is available by application under the provisions of the Australian Bureau of Statistics (ABS).
